# Network toxicology and single-cell analysis reveal molecular mechanisms of DEHP-induced colorectal cancer

**DOI:** 10.6026/973206300223430

**Published:** 2026-06-30

**Authors:** Yingyi Wei, Weixue Li, Wenhua Huang, Zhai Cai

**Affiliations:** 1 School of Basic Medical Sciences, Dali University, School of Fundamental Sciences, Dali University, Dali, Yunnan Province, China; 2 Department of Nursing, West China Hospital, West China Hospital of Sichuan University, Chengdu, Sichuan, China; 3 Department of Basic Medicine, Southern Medical University, Southern Medical University Second Clinical Medical College, Guangzhou, Guangdong, China; 4 Department of General Surgery, West China Hospital, Zhujiang Hospital, Southern Medical University, 253 Xingang East Road, Haizhu District, Guangzhou 510282, Guangdong, China

**Keywords:** Network toxicology, di(2-ethylhexyl) phthalate (DEHP), colorectal cancer (CRC), machine learning (ML), molecular docking.

## Abstract

Di(2-ethylhexyl) phthalate (DEHP) is a common environmental pollutant associated with colorectal cancer (CRC), but its molecular mechanisms remain unclear. Hence, we integrated toxicological target screening, weighted gene co-expression network analysis (WGCNA), and 113 machine learning algorithms to construct a diagnostic model for DEHP-related CRC. Single-cell transcriptomics and molecular docking were further applied to analyze the cellular distribution of key genes and the interactions between DEHP and target proteins. Five core genes (MMP7, CDK4, MMP1, CNR1, and TNFRSF1A) were identified and linked to inflammation, cell cycle regulation, and PI3K-Akt signaling. Thus, we report the molecular basis of DEHP-induced CRC progression providing a novel strategy for identifying environmental carcinogenic biomarkers and therapeutic targets.

## Background:

DEHP, widely used in plastics, is a persistent pollutant with <10% recycling rate, resisting degradation and accumulating in ecosystems [1]. DEHP can spread through food chains, air and water, resulting in detectable residues in crops, soil and marine organisms, thereby increasing potential risks to human health through dietary exposure [2]. Long-term exposure to DEHP has been associated with reproductive toxicity, developmental abnormalities, and cancer-related biological alterations [3]. Although direct epidemiological evidence linking DEHP exposure to colorectal cancer (CRC) remains limited, experimental studies suggest that DEHP may contribute to colorectal carcinogenesis Error! Reference source not found. DEHP has been reported to activate MDR1 expression and to promote DMH-induced colon tumor formation in rat models [5]. In addition, hthalate exposure may promote colorectal cancer progression by activating the PI3K/AKT and Wnt/β-catenin signaling pathways, which are closely associated with tumor migration and invasion [6]. Therefore, it is of interest to describe the molecular mechanisms and potential targets involved in DEHP-induced colorectal cancer by integrating toxicological target identification, differential expression analysis, functional enrichment analysis, machine learning modeling, single-cell analysis, and molecular docking approaches.

## Methods and Materials:

## Data acquisition:

DEHP toxicological targets were retrieved from ChEMBL, SwissTargetPrediction, and SEA. Six CRC-related microarray datasets from GEO were used: GSE10950, GSE25070, and GSE110224 served as the training set (each with matched normal and tumor samples), while GSE32323, GSE41258, and GSE41328 constituted the validation cohort to ensure model robustness.

## Weighted gene co-expression network analysis:

We constructed gene co-expression networks using the WGCNA package in R. Duplicate probes were merged using the avereps function, and genes with standard deviation > 0.5 were retained. Hierarchical clustering (Euclidean distance, average linkage) was used to detect outlier samples at a cut height of 20,000. The soft-thresholding power β was determined using the pick Soft Threshold function (scale-free topology fit index R^2^≈ 0.8) Error! Reference source not found. The adjacency matrix was transformed into a topological overlap matrix (TOM), and genes were clustered based on the 1-TOM distance. Modules were identified using dynamic tree cutting (deep Split = 2, minimum module size = 60) and merged when module eigengene correlations exceeded 0.75 (cut Height = 0.25). Module-trait relationships were assessed using Pearson correlation, and p-values were calculated using the Student t-test. Module membership (MM) and gene significance (GS) were also calculated.

## Identification of toxicological targets and GO/KEGG analysis:

Differentially expressed genes (DEGs) between CRC and normal samples were identified using limma (|log_2_FC| > 1, adjusted p < 0.05) Error! Reference source not found.. Overlapping genes between DEHP toxicological targets and DEGs highlighted candidate mediators of DEHP-induced CRC. These targets underwent GO (MF, CC, BP) and KEGG enrichment via clusterProfiler to identify potential signaling pathways driving the toxicant's effects, with results ranked by gene count and p-value Error! Reference source not found..

## Core gene identification via machine learning:

Thirteen machine learning algorithms (Stepglm forward/both/backward, Enet across α values, GBM, glmBoost, Naive Bayes, Lasso, SVM, XGBoost, Ridge, plsRglm, Random Forest, LDA) were applied to the training data for feature selection and CRC diagnostic model building. After internal training, each model was externally validated. The model achieving the highest average area under the curve (AUC) across both datasets was selected to prioritize core genes implicated in DEHP-induced CRC.

## Single-cell sequencing analysis:

Single-cell RNA-seq data (GSE231559) comprising five patient groups and three controls were processed with Seurat for QC, dimensionality reduction, and clustering. Harmony corrected batch effects Error! Reference source not found., Single R provided cell-type annotation and Clustree visualized cluster relationships Error! Reference source not found.. FeaturePlot, VlnPlot, and DotPlot functions displayed expression patterns of hub genes across identified cell populations to validate their cell-type specificity [12].

## Molecular docking:

Core protein structures were downloaded from the PDB, cleaned in PyMOL (removing ligands and water, adding hydrogens, and identifying rotatable bonds), and converted to pdbqt format. AutoDock Vina 1.2.0 performed docking with DEHP (exhaustiveness = 25), retaining the top nine conformations; binding free energies were computed using the Lamarckian genetic algorithm. PyMOL was used to visualize protein-DEHP complexes and annotate binding energies and key interacting residues.

## Results:

We integrated the GEO datasets and normalized the gene expression matrix after removing batch effects. Principal component analysis (PCA) showed significant improvement in the normalized data (Figure 1A-B - see PDF). The volcano plot and heatmap revealed the differential expression patterns of these genes (Figure 1C-D - see PDF). We performed weighted gene co-expression network analysis (WGCNA) using the "WGCNA" package. The soft threshold was set to 14, and the fitting index (R^2^) was 0.86 (Figure 2D - see PDF). WGCNA analysis identified eight co-expression modules, among which the blue module exhibited the strongest correlation with CRC (cor = 0.84, P = 3e-36) and was selected as the target module. The blue module contained 1,942 genes (Figure 1E - see PDF). We then integrated the differentially expressed genes with the target module genes, and after removing duplicates, we obtained a total of 2,590 CRC-related genes (Figure 1F - see PDF). A total of 2,590 CRC-related genes were obtained by integrating the DEGs (n = 648) and two WGCNA module genes (n = 659 and n = 1,283), taking the union of the three sets and removing duplicates. These genes were subsequently used for intersection analysis with DEHP toxicological targets (Figure 1G - see PDF).

We obtained 211 DEHP-related toxicological targets from ChEMBL (https://www.ebi.ac.uk/chembl/), SwissTargetPrediction (http://swisstargetprediction.ch/) and SEA (SEA Search Server (bkslab.org)). After intersecting these targets with CRC-related genes, we identified 24 potential toxicological targets involved in DEHP-induced CRC (Figure 2A-B - see PDF). We then constructed a network diagram of the hub toxicological targets (Figure 2-D - see PDF). Additionally, we performed Gene Ontology (GO) and Kyoto Encyclopedia of Genes and Genomes (KEGG) analyses on these potential toxicological targets to explore the molecular mechanisms underlying DEHP-induced CRC. The GO results were primarily enriched in pathways related to the regulation of the inflammatory response, the positive regulation of the mitotic cell cycle, and cell cycle phase transitions. The KEGG analysis revealed key pathways including neuroactive ligand-receptor interaction, PI3K-Akt signaling pathway, calcium signaling pathway, cGMP-PKG signaling pathway, and cell cycle-related pathways.

We constructed a diagnostic model for CRC patients based on the results shown in the figures. To optimize its performance; we combined 113 modeling algorithms and selected the best AUC across both the training and validation groups. The Random Forest (RF) algorithm achieved the highest average AUC value (0.997) in the training group, identifying 11 key genes induced by DEHP in CRC (Figure 3A - see PDF). To enhance the interpretability of the model, we performed SHAP analysis to assess the contribution of each gene to the model. The top five genes with the highest contributions were selected as our core genes for further analysis. The diagnostic potential of these core genes was validated using ROC curve analysis, with all five genes showing AUC values greater than 0.80 (Figure 3B - see PDF). The volcano plot displayed the differential expression patterns of the core genes in CRC patients (Figure 3C - see PDF). Global importance analysis revealed that MMP7 had the highest average SHAP value, indicating its greatest contribution to the model (Figure 3D - see PDF). The SHAP beeswarm plot further illustrated the contribution patterns of the five core genes. High expression of MMP7, CDK4, and MMP1 showed positive contributions, whereas high expression of CNR1 and TNFRSF1A showed negative contributions (Figure 3E - see PDF). The SHAP beeswarm plot showed the contribution of each gene to the model's prediction. Among the five genes of interest, MMP7, CDK4, and MMP1 positively influenced the model at higher expression levels, while CNR1 showed negative regulation at lower expression levels (Figure 3F - see PDF). In the representative individual's force-directed plot, MMP7 had the most significant negative impact on the model (-0.172), while CDK4 and CNR1 contributed smaller negative effects. After combining all features, the model's predicted value f(x) = 0.0314 was significantly lower than the baseline value E[f(x)] = 0.503, indicating that these gene features had a significant impact on the prediction result for this sample (Figure 3G - see PDF)

We utilized single-cell transcriptomic data from CRC patients in the GEO database to explore the expression of core genes. First, clustering analysis was performed, and the results were annotated by cell type (Figure 4A-B - see PDF). Then, a bubble plot was used to show the expression distribution of the core genes across different cell types.CDK4 and TNFRSF1A were significantly enriched in fibroblasts, whereas MMP7 was mainly expressed in epithelial cells (Figure 4C - see PDF). Additionally, UMAP and bar plots further displayed the expression and distribution of core genes across different cell types (Figure 4D-E - see PDF). These results revealed the specific distribution of these core genes across different cell subgroups in colon tissues, providing important insights into their role in the pathogenesis of CRC.

To verify potential interactions between DEHP and the core proteins, we conducted a comprehensive molecular docking analysis. The results showed that DEHP exhibited strong binding affinity for all the proteins, with binding affinities all less than 0 kcal/mol, indicating that the binding occurs spontaneously without the need for external energy (Table 1). Visualization of the binding conformations revealed that all proteins formed multiple chemical bonds with DEHP, suggesting that the stability of the complexes is reliable (Figure 5A-E - see PDF). These findings provide structural evidence of direct molecular interactions between DEHP and the core targets identified by machine learning.

## Discussion:

Colorectal cancer (CRC) is a major global digestive malignancy driven by interactions between genetic susceptibility and environmental exposures. Among these environmental factors, the plasticizer di(2-ethylhexyl) phthalate (DEHP) has attracted increasing attention because of its endocrine-disrupting and potential carcinogenic properties [13]. DEHP can enter the human body through diet, inhalation, and dermal exposure, accumulate in the intestine, and potentially contribute to CRC initiation. However, the molecular mechanisms underlying DEHP-associated colorectal carcinogenesis remain poorly understood Error! Reference source not found.. Using network toxicology, single-cell transcriptomics, and multi-algorithm machine learning, this study delineates the DEHP-CRC molecular network. A total of 211 potential DEHP targets were identified from the Swiss Target Prediction, SEA, and ChEMBL databases, and an intersection with CRC-related genes yielded 24 candidate targets. Further screening using multiple machine learning models, combined with SHAP feature importance analysis and identified five key genes: MMP7, MMP1, CDK4, TNFRSF1A, and CNR1. Single-cell expression analysis revealed that these genes were expressed across multiple colorectal cell types. The relatively high expression of CDK4 and TNFRSF1A suggests their potential involvement in tumor-associated epithelial and stromal compartments [15]. In addition, molecular docking analysis demonstrated strong binding affinity between DEHP and these proteins, with binding energies below 0 kcal/mol. This indicates that DEHP may directly interact with these targets and influence their biological functions. Gene Ontology enrichment analysis indicated that the characteristic genes were mainly associated with regulation of inflammatory response, cyclin- dependent protein kinase complex, and serine-type endopeptidase activity. These findings suggest that DEHP exposure may contribute to tumorigenesis through inflammatory activation, cell cycle dysregulation, and extracellular matrix remodeling. DEHP and its metabolite MEHP impair intestinal barrier integrity and induce pro-inflammatory cytokines, thereby creating a tumor-favorable microenvironment. In addition, DEHP has been shown to enhance CRC cell proliferation and chemotherapy resistance through upregulation of CDK-related genes and metabolic alterations. The enrichment of serine-type endopeptidase activity further suggests enhanced protease-mediated extracellular matrix degradation. KEGG pathway analysis also revealed significant enrichment in neuroactive ligand-receptor interaction and the p53 signaling pathway. This indicates that DEHP may disrupt neuroimmune regulation and genomic stability through oxidative stress, DNA damage, or inhibition of p53-mediated apoptosis. These findings suggest that DEHP exposure may promote CRC progression through multiple mechanisms: inflammation, cell-cycle imbalance, matrix remodeling, and p53 pathway disruption [16]. In CRC, MMP7 is frequently overexpressed in epithelial cells and acts as a downstream target of Wnt/β-catenin signaling, thereby promoting tumor cell migration and metastasis [17]. In contrast, MMP1 is mainly produced by tumor cells or stromal fibroblasts and degrades type I and III collagen, which is closely associated with tumor invasion and poor prognosis. Toxicological studies show that DEHP and MEHP activate multiple signaling pathways, including Akt, NF-kB, and Wnt, promoting epithelial-mesenchymal transition and tumor stemness. These signaling changes may increase MMP7 and MMP1 expression, forming a mechanistic axis linking environmental exposure, pathway activation, matrix degradation, and tumor invasion. Therefore, DEHP exposure may indirectly enhance MMP7- and MMP1- mediated matrix degradation and tumor infiltration through oxidative stress and inflammatory signaling pathways. CDK4 is a key regulator of the G1-S phase transition in the cell cycle [18]. When forming a complex with cyclin D, CDK4 phosphorylates the retinoblastoma protein, releasing E2F transcription factors and promoting DNA replication and cell proliferation. In CRC, CDK4 is frequently overexpressed and contributes to sustained cell proliferation, cell cycle checkpoint evasion, and chemotherapy r esistance. Previous studies suggest that DEHP exposure activates Akt/ERK signaling, increasing cyclin D1/CDK4 expression and accelerating S-phase entry, thereby promoting abnormal cell proliferation [19]. Cannabinoid receptor 1 (CNR1) encodes a G protein-coupled receptor that is a central component of the endogenous cannabinoid system Error! Reference source not found. In the gastrointestinal tract, CNR1 participates in the regulation of appetite, energy balance, intestinal motility, and inflammatory responses. Increasing evidence suggests that CNR1 signaling can inhibit tumor cell proliferation and induce apoptosis [21]. DEHP and its metabolite MEHP have been reported to activate peroxisome proliferator-activated receptor gamma (PPAR gamma), which can regulate CNR1 transcription. Therefore, DEHP exposure may alter CNR1 expression through PPAR gamma activation and consequently disturb intestinal homeostasis. Tumor necrosis factor receptor superfamily member 1A TNFRSF1A mediates signaling of the tumor necrosis factor pathway and plays a crucial role in inflammation and apoptosis regulation. In CRC, activation of TNFRSF1A signaling can promote tumor cell survival and proliferation and contribute to remodeling of the tumor microenvironment. DEHP-induced inflammation may increase TNFRSF1A expression by activating the NF-κB pathway, amplifying inflammatory responses and promoting epithelial-mesenchymal transition and tumor invasion. Furthermore, molecular docking results showing binding energies below 0 kcal/mol further support the possibility that DEHP may directly interact with these proteins [22]. Although this study integrates network toxicology and machine learning to identify potential targets and pathways linking DEHP exposure to CRC, several limitations should be noted. The predicted interactions between DEHP and candidate targets, as well as the biological functions of the identified core genes, still require experimental validation. In addition, the datasets used in this study were derived from public databases, and potential heterogeneity among datasets may influence the robustness of the results. Furthermore, transcriptomic analysis cannot fully capture the complexity of the tumor microenvironment, and additional validation in cell-based or animal models will be necessary in future studies.

## Conclusion:

We integrated network toxicology with multi-algorithm machine learning to identify five key genes potentially involved in DEHP-related colorectal carcinogenesis. Molecular docking suggests that these genes may directly interact with DEHP and disrupt related signaling networks. This work offers a novel in silico framework integrating network toxicology and machine learning to decode DEHP-driven CRC mechanisms. It addresses a key gap in linking environmental plasticizers to colorectal carcinogenesis. While the findings require experimental validation, they lay a foundation for future *in vivo*, cohort, and mechanistic studies to confirm these predictive biomarkers and therapeutic targets.

## Figures and Tables

**Table 1:** DEHP- core gene molecular docking information

**Protein**	**PDB ID**	**Binding energy (kcal/mol)**
CDK4	2W96	-6.1
CNR1	5TGZ	-5.5
MMP1	1CGE	-6
MMP7	1MMQ	-6.5
TNFRSF1A	1F3V	-4.5
